# Case Report: Recognizing Pneumatosis Intestinalis: A Case of Bowel Ischemia Presenting as Renal Colic

**DOI:** 10.1155/2012/575342

**Published:** 2012-02-16

**Authors:** Peter D. Corr

**Affiliations:** Department of Radiology, Faculty of Medicine, United Arab Emirates University, P.O. Box 17666, Al Ain, United Arab Emirates

## Abstract

The clinical diagnosis of bowel ischemia is often difficult and the diagnosis can easily be missed unless there is a high index of clinical and radiological suspicion. Bowel ischemia and or infarction must be considered in the differential diagnosis in the older patient with pre-existing coronary artery or generalized vascular disease, cardiac failure, or arrhythmias especially atrial fibrillation and hypertension. An elderly patient with caecal infarction is presented who was initially diagnosed and treated for renal colic.

## 1. Introduction

The clinical diagnosis of bowel ischemia is often difficult because the symptoms and signs of abdominal pain and tenderness on palpation are often nonspecific in nature [1]. The diagnosis can easily be missed unless there is a high index of clinical and radiological suspicion when dealing with a patient with acute abdominal pain [2]. Bowel ischemia and or infarction must be considered in the differential diagnosis in the older patient with pre-existing coronary artery or generalized vascular disease, cardiac failure, or arrhythmias especially atrial fibrillation and hypertension. An elderly patient with caecal infarction is presented who was initially diagnosed and treated for renal colic.

## 2. Case Report

**Figure 1:**
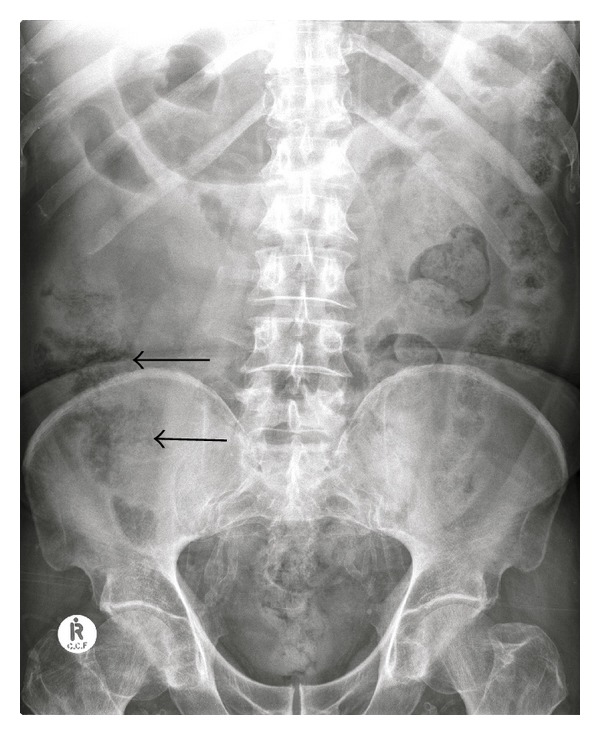
Supine abdomen demonstrates extensive pelvic vascular calcification. However no radio-opaque calculi are present. The bowel gas pattern was initially considered to be normal, but closer inspection demonstrates pneumatosis intestinalis within the cecal wall (arrow).

**Figure 2:**
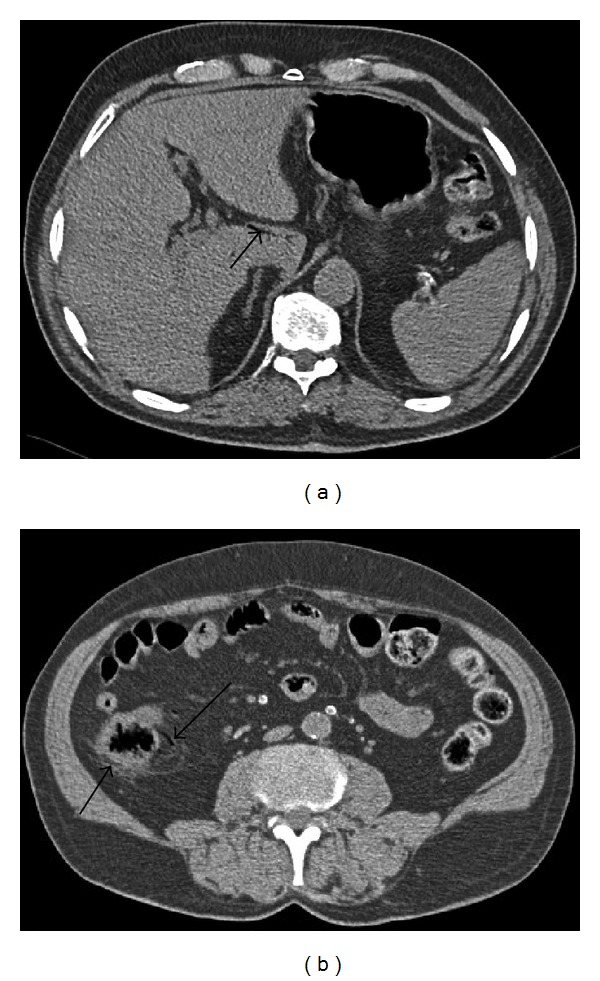
(a) Noncontrast CT image of the liver demonstrates intraluminal gas in a portal venule in the caudate lobe (arrow). (b) Noncontrast CT image through the lower abdomen demonstrates cecal wall thickening with fat stranding and linear pneumatosis intestinal within the cecal wall with intraluminal gas in adjacent mesenteric venules (arrows). Note the calcified mesenteric artery branches.

A 71-year-old man with a known history of end-stage diabetic nephropathy and hypertension presented to the emergency room with a history of acute right abdominal pain for six hours. The pain was localized in the right loin, colicy in nature, and radiated to the right inguinal region. He was on the chronic hemodialysis program for chronic renal failure and had just completed his last dialysis eight hours earlier. Examination demonstrated an elderly man in some distress with right loin tenderness and guarding. Blood pressure and pulse were within normal limits for his age. Laboratory results were normal except for an elevated C reactive protein level and serum creatinine. His hemoglobin and the white cell count were within normal limits. A working diagnosis of right renal colic was made and an urgent supine abdominal radiograph and non enhanced CT scan of the kidneys, ureters, and bladder (KUB) were requested to confirm the clinical diagnosis. The supine abdominal radiograph (Figure 1) demonstrated extensive arterial calcification, what was initially considered to be a “normal bowel gas” pattern but no radio-opaque calculi were detected. A nonenhanced CT KUB scan was performed to detect ureteric obstruction and hydronephrosis from renal or ureteric calculi. On the CT study small quantities of linear gas were detected in the peripheral portal vein branches of the caudate lobe of the liver (Figure 2(a)), the peripheral mesenteric veins branches to the caecum and ascending colon, and a thickened wall of cecal wall with pericecal fat stranding (Figure 2(b)). Calcification of the walls of the superior and inferior mesenteric arteries was noted. Both kidneys were small with cortical thinning but there were no renal or ureteric calculi detected. There was no hydronephrosis or ureteric dilatation present. A radiological diagnosis of cecal and ascending colon infarction was made. The patient was taken to theatre for laparoscopy which confirmed patchy cecal wall infarction. A right hemicolectomy was then performed with a primary anastomosis. Transmural necrosis of the cecal wall was confirmed on histology. The patient had a stormy postoperative course but has since recovered from his surgery 4 months ago.

## 3. Discussion

The preoperative diagnosis of cecal wall infarction was made on the noncontrast CT study from the detection of small quantities of gas within the cecal wall (pneumatosis intestinalis), mesenteric vein intraluminal gas and intrahepatic portal vein gas. With the presence of extensive diabetic vascular disease, the patient is thought to have suffered a non-occlusive mesenteric infarct of the caecum following a hypotensive episode during the most recent dialysis procedure prior to this presentation. Linear gas in the bowel wall in the presence of portal and mesenteric venous gas with associated bowel wall thickening is especially concerning for bowel wall infarction and must be communicated immediately to the referring physician. This sign is a “red flag” for bowel wall ischemia and infarction. Gas in the intrahepatic portal vein branches is often detected on CT in the periphery of the liver and must be differentiated from central perihilar gas found in the biliary tree [3]. Bowel infarction must be considered in older patients in the presence of cardiovascular disease, sepsis, neutropenic colitis or toxic megacolon [4]. “Benign-” type pneumatosis intestinalis is more often “cystic” or “bubbly” in appearance. “Benign-” type pneumatosis intestinalis is detected in the presence of chronic obstructive pulmonary diseases, in patients on positive pressure ventilation, with collagen vascular disease, after endoscopy or surgical bowel anastomoses, in patients on chronic immunotherapy, chemotherapy, or corticosteroid treatment [4].

Mesenteric infarction can be due to mesenteric arterial or venous occlusion. Arterial occlusion follows an embolus in the proximal 3–8 cm of the superior mesenteric artery or thrombosis usually at the first branch of the artery [5]. Thrombosis in the mesenteric veins is usually due to a hypercoagulable disorder [6]. Mesenteric arterial infarction may be due to nonocclusive hypoperfusion in patients with cardiac failure and or mesenteric arterial vascular disease following an episode of hypotension or shock as presumably experienced by this patient.

CT is the most sensitive examination to detect pneumatosis intestinalis. CT angiography (CTA) has the advantage of demonstrating the patency of the coeliac and mesenteric arteries noninvasively on a contrast CT study [7]. This patient had only a limited CT study to detect renal or ureteric calculi and the detection of pneumatosis intestinalis on the study was an incidental but extremely important finding. This case demonstrates the importance of considering bowel wall infarction in the differential diagnosis of any high-risk patient presenting with acute abdominal pain.
